# Quality of seclusion medical review according to trust guidelines

**DOI:** 10.1192/bjo.2021.585

**Published:** 2021-06-18

**Authors:** Shumaila Shahbaz, Richard Ward

**Affiliations:** Humber Teaching Foundation Trust

## Abstract

**Aims:**

We accessed whether medics are following Trust Policy while conducting seclusion medical review and identify the strengths in quality of seclusion medical review and identify the areas which need improvements to improve our quality and standards of patient's care and safety and to reduce risks.

**Background:**

The Mental Health Act Code of Practice sets an expectation for mental health services for restrictive interventions (use of restraint, seclusion and rapid tranquilisation) by following good standards. Medical reviews provide an opportunity to evaluate and amend seclusion management plan. This clinical audit was undertaken by looking at quality of record keeping about seclusion review by junior doctors, staff grades and consultants at different times (day, night, and weekend).

**Method:**

Data analysis was carried out by using Microsoft Excel. The audit had Humber Teaching NHSFT approval. We assessed electronic healthcare records. Data collection was carried out or retrospectively in 2019(n = 40) using following parameters:

1. A review of patient's physical and psychiatric health.
2. An assessment medication prescribed and adverse effects of medication.
3. A review of observations required.
4. An assessment of the risk posed by the patient to others.
5. An assessment of any risk to the patient from deliberate or accidental self-harm.
6. An assessment of need for continuing seclusion, and whether it is possible for seclusion measures to be applied more flexibly, or in a less restrictive manner.
7. Time of Seclusion Review: Within first hour after seclusion and then every 4 hours until internal MDT. After MDT twice a day.
8. Record Keeping.

**Result:**

Key Successes (above 80%)

Time of seclusion review (with in first hour or when required)

Record keeping (accurate time and place for clinical notes).

Plan for continuing need for seclusion.

Good documentation of Risk to self and risk to others.

Good documentation of mental state examination.

Comments on physical health although it can be improved.

Key Concerns(Less than 60%):

Prescribed Medications.

Medication side effects.

Physical Observations

**Conclusion:**

Medics are missing some important parts in seclusion medical review. We developed a template for seclusion medical review according to trust guidelines which are based on Code of Practice and to incorporate in already existing seclusion review form. We also delivered teaching and training to doctors and also showed junior doctor's an example of documentation. We will re-audit in 1 years’ time to see improvement.

